# Supplementary material for: Physical Activity-Related Injuries and Risk Factors among Secondary School Students in Hong Kong
Source: Int J Environ Res Public Health. 2020 Jan 24;17(3):747. doi: 10.3390/ijerph17030747 (PMC7036798; doi:10.3390/ijerph17030747)
Supplement: Supplementary file 1 [file ijerph-17-00747-s001.pdf]

Supplementary Table S1. Distribution of PA level by sex and school.

Variables	All	School A	School B	School C
**BOYS**				
MVPA level (min/week)				
Median	400.00	390.00	385.00	440.00
25th percentile	210.00	180.00	200.00	0.00
75th percentile	680.00	765.00	597.50	270.00
Mean	519.73	539.26	470.15	554.38
Standard deviation	473.35	539.57	397.89	466.77
VPA level (min/week)				
Median	150.00	155.00	90.00	180.00
25th percentile	30.00	60.00	260.00	60.00
75th percentile	360.00	360.00	710.00	360.00
Mean	233.74	276.51	178.88	254.84
Standard deviation	277.06	341.36	233.03	238.01
**GIRLS**				
MVPA level (min/week)				
Median	340.00	300.00	360.00	380.00
25th percentile	180.00	150.00	200.00	210.00
75th percentile	600.00	495.00	606.25	703.75
Mean	453.00	398.61	463.28	504.56
Standard deviation	411.15	424.18	395.209	410.40
VPA level (min/week)				
Median	89.00	90.00	60.00	90.00
25th percentile	0.00	43.75	0.00	0.00
75th percentile	240.00	245.00	195.00	292.50
Mean	162.25	174.73	132.46	195.45
Standard deviation	217.32	228.46	191.495	237.51
